# Neonatal outcomes when intravenous esketamine is added to the parturients transferred from labor analgesia to emergency cesarean section: a retrospective analysis report

**DOI:** 10.1186/s12871-023-02132-x

**Published:** 2023-05-17

**Authors:** Zhaojia Liang, Ting Zhou, Mengxia Wang, Yalan Li

**Affiliations:** Department of Anesthesiology, The First Affiliated Hospital of Jinan University, Guangzhou, 510630 China

**Keywords:** Esketamine, Cesarean section, Epidural anesthesia, Neonatal outcomes

## Abstract

**Objectives:**

The use of intravenous analgesics during emergency cesarean section may lead to adverse neonatal outcomes. In our study, we investigated whether a single intravenous (i.v.) dose of 25 mg esketamine administered to parturients with inadequate analgesia during epidural anesthesia for cesarean section would affect the neonate.

**Design:**

We reviewed the records of parturients who were transferred from labor analgesia to epidural anesthesia for emergency cesarean section from January 2021 to April 2022. Parturients were grouped by whether they received esketamine infusions during the incision–delivery interval. Neonatal outcomes, including umbilical arterial-blood gas analysis (UABGA), Apgar score, and total days spent by the neonate in the hospital, were compared between the two groups. The secondary outcomes of this study included BP, heart rate (HR), SPO_2_ and the incidence of adverse effects in parturients during operation.

**Setting:**

China.

**Results:**

After propensity score matching, 31 patients remained in each of the non-esketamine and esketamine groups. There were no significant differences in neonatal outcomes, including UABGA, Apgar score, and total days in the hospital, between the two groups. Additionally, our study showed a similar hemodynamic performance in parturients between the two groups during operation.

**Conclusions:**

Intravenous esketamine (25 mg) is safe for neonates when it is given to parturients transferred from labor analgesia to emergency cesarean section.

## Introduction

Lumbar epidural is the most efficient method for relieving pain in delivery. When the parturient fails to deliver her neonate and needs an emergency operation, epidural anesthesia (EA) is the most common approach, in which an epidural catheter is inserted into the epidural space to give the parturient labor analgesia. Cesarean section, one of the most common in-hospital surgeries, has significantly reduced neonatal mortality and saved millions of new mothers [1]. However, spinal anesthesia is accompanied by a high incidence of intraoperative pain. An article published in 2016 described a patient’s intraoperative pain during a cesarean section under spinal anesthesia [2]. She reported that her pain resulted in psychological and physical problems for a long period of time. To relieve such terrible pain, the anesthesiologist has to use anesthetic drugs on the parturients.

Ketamine is an anesthetic agent with analgesic effect. As it prevents central sensitization and abolishes peripheral-afferent noxious stimuli, [3, 4] it has an excellent analgesic effect and is widely used in clinical practice. It is reported that ketamine can reduce visceral-traction pain from surgical interventions and relieve tension in parturients undergoing cesarean section without any adverse effects [5]. Therefore, it is an ideal choice of analgesic for parturients suffering from inadequate analgesia, even those with severe fetal distress [6, 7]. Esketamine is an S + isomer of ketamine with similar pharmacological properties.

There is little information available about the effect of ketamine isomers on the neonate through the placenta when it is administered to pregnant women undergoing cesarean section; more research is needed to fully analyze such effect. In our study, we investigated whether a single intravenous (i.v.) dose of 25 mg esketamine administered to parturients with inadequate analgesia during EA for cesarean section would affect the neonate.

## Materials and methods

This was a retrospective investigative study performed at a single center. The study was approved by the Human Ethics Committee of the First Affiliated Hospital of Jinan University (No. KY-2022-129). Given the retrospective design, written informed consent was waived by the institutional review board.

Subjects were pregnant women of American Society of Anesthesiologists (ASA) status I and II, age ≥ 18 years, at ≥ 37 weeks of gestation, who were transferred from labor analgesia to EA for emergency cesarean section from January 2021 to April 2022.

Exclusion criteria were as follows: placenta previa, placental abruption, umbilical cord around the neonate’s neck, infants of low birth weight (≤ 2500 g), gestational hypertension, and eclampsia.

All parturients had been given labor analgesia, and an epidural catheter was inserted into the epidural space through the L3–4 interspace. Electrocardiography (ECG), non-invasive blood pressure (BP) and pulse oximetry (SPO_2_) were continuously monitored.

All parturients received EA with 1% ropivacaine(Naropin, AstraZeneca AB,Sweden), 10–15 ml. During the incision–delivery interval, if a parturient still felt pain, she was given a single i.v. dose of 25 mg esketamine(Esketamine, Jiangsu Hengrui,China). This administration was performed within five minutes after the operation. We grouped parturients by whether they received esketamine infusions.

Immediately after delivery (within 60s after birth), the obstetrician double clamped the umbilical cord and collected 1ml of blood from the umbilical artery attaching the fetal. Blood gas analysis was performed using an ABL80 FLEX blood gas analyzer (Radiometer, Brønshøj, Denmark) right away. Primary outcomes included pH, base excess (BE), and lactate levels. In addition, we assessed the incidence of fetal acidosis, defined as BE greater than − 6 mM. The pediatrician in attendance recorded Apgar scores at 1, 5, and 10 min. Total days that neonates spent in the hospital were also recorded. The secondary outcomes of this study included BP, heart rate (HR), SPO_2_ and the incidence of adverse effects in parturients during operation. Maternal characteristics including age, height, weight, gestational age, fetal weight, duration of labor (from regular uterine contractions to the birth of neonate), stage of labor (when the parturient was sent to operating room), oxytocin administration and dose of ropivacaine were recorded at the same time.

All patient information was obtained from the electronic medical record system (Mandala T Software Corp., Wuxi, China) and the Mediston Anesthesia Information System (Mediston Medical Technology Co., Suzhou, China).

### Statistical analysis

We used propensity score matching (PSM) to match the two groups in order to adjust for differences in demographics. Patients were matched as 1:1 pairs using nearest-neighbor (NN) matching of propensity score (PS). We conducted PSM with the following variables: age, height, weight, gestational age, fetal weight, duration of labor, stage of labor, oxytocin administration and dose of ropivacaine. PSM was performed using R software version 4.2.2 (R Foundation for Statistical Computing, Vienna, Austria).

Continuous data were expressed as means ± standard deviations (SDs), and counting data as frequencies and percentages. We used the Shapiro–Wilk test to test the normality of data and Levene’s test to test their homogeneity. An independent-sample *t* test was used to test normally distributed data, and the Mann–Whitney *U* test was applied to non-normally distributed data. We compared categorical data using a *χ*^2^ test or Fisher’s exact test. Repeated-measurement data were analyzed by a repeated measures analysis of variance. Statistical significance was defined as *P* < 0.05. We performed all statistical analyses using SPSS version 29.0.0.0(241) (IBM Corp., Armonk, NY, USA).

## Results

**Fig. 1:**
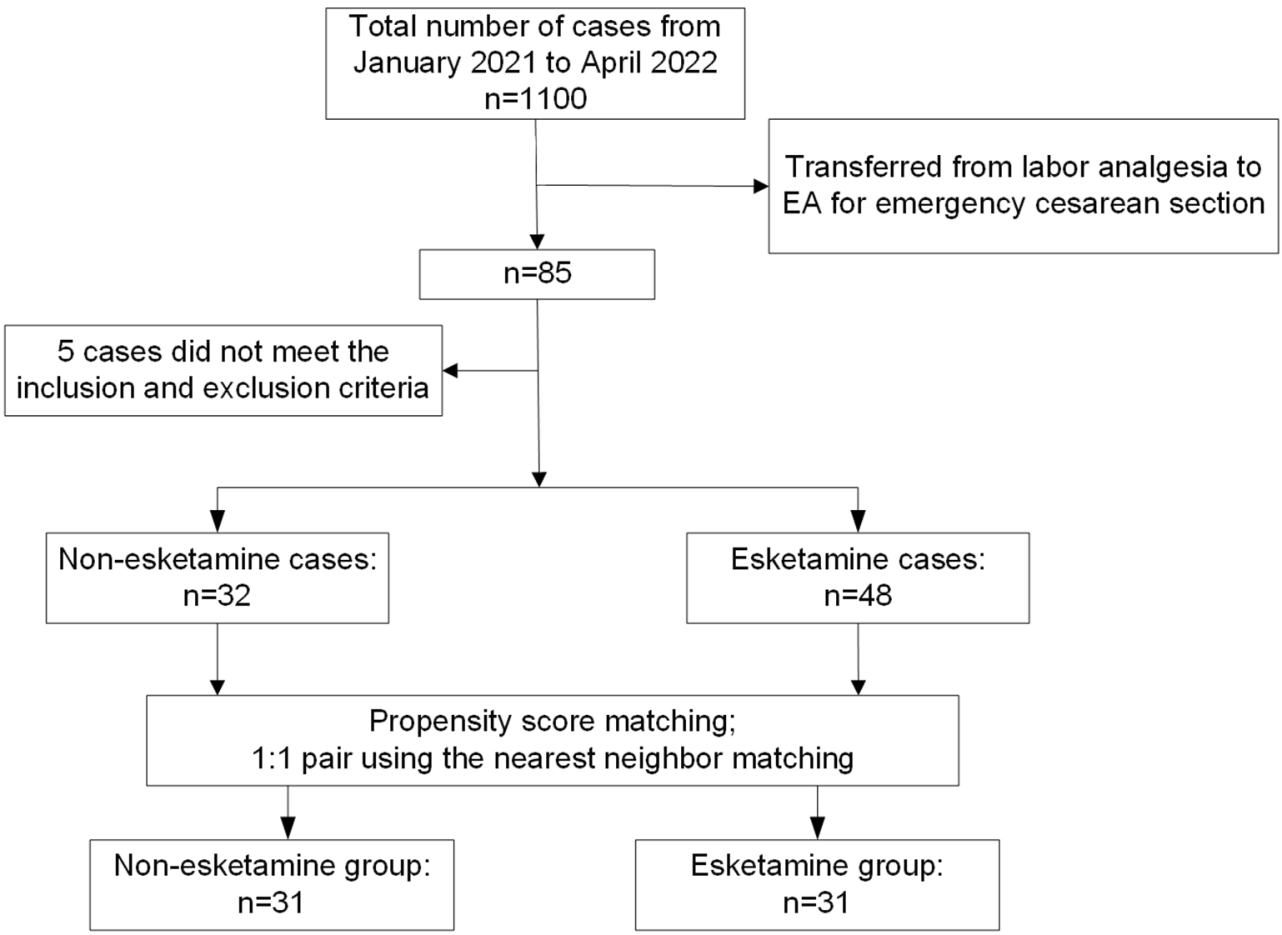
The flow chart of patient enrollment

From January 2021 to April 2022, 1100 patients underwent cesarean section; 85 of them were transferred from labor analgesia to EA for emergency cesarean section. After we applied inclusion and exclusion criteria, 32 patients remained in the non-esketamine group, and 48 in the esketamine group. After PSM, 31 patients remained in each of the two groups (Fig. 1). The demographics of the original cohort and the matched cohort are shown in Table 1.

**Table 1:** Demographic characteristics

	Original cohort		*P*	Matched cohort		*P*	SMD	
	Non-esketamine	Esketamine		Non-esketamine	Esketamine			
Age (years)	32.3 ± 4.0	30.6 ± 3.2	0.039	32.3 ± 4.0	31.3 ± 2.9	0.304	-0.399	
Weight (kg)	65.3 ± 8.6	68.2 ± 11.3	0.634	65.3 ± 8.8	65.4 ± 7.7	0.936	0.022	
Height (cm)	156.2 ± 4.9	158.8 ± 5.1	0.027	156.1 ± 5.0	156.9 ± 4.7	0.482	0.184	
Gestational age (days)	279.1 ± 5.4	279.5 ± 5.6	0.738	279.0 ± 5.4	279.4 ± 5.5	0.756	0.075	
Fetal weight (kg)	3.3 ± 0.2	3.2 ± 0.3	0.441	3.3 ± 0.2	3.3 ± 0.3	0.789	-0.058	
Duration of labor(min)	320.8 ± 84.5	315.0 ± 84.5	0.785	319.2 ± 85.5	307.0 ± 86.4	0.578	0.003	
Stage of labor			0.731			1.000	0.000	
1	93.7%	91.6%		93.5%	93.5%			
2	6.3%	8.4%		6.5%	6.5%			
oxytocin administration	71.8%	66.7%	0.625	74.1%	70.9%	0.778	-0.071	
Dose of ropivacaine(mg)	13.9 ± 2.1	13.5 ± 2.2	0.471	13.8 ± 2.1	13.7 ± 2.2	0.769	-0.072	

SMD = standardized mean difference

**Fig. 2:**
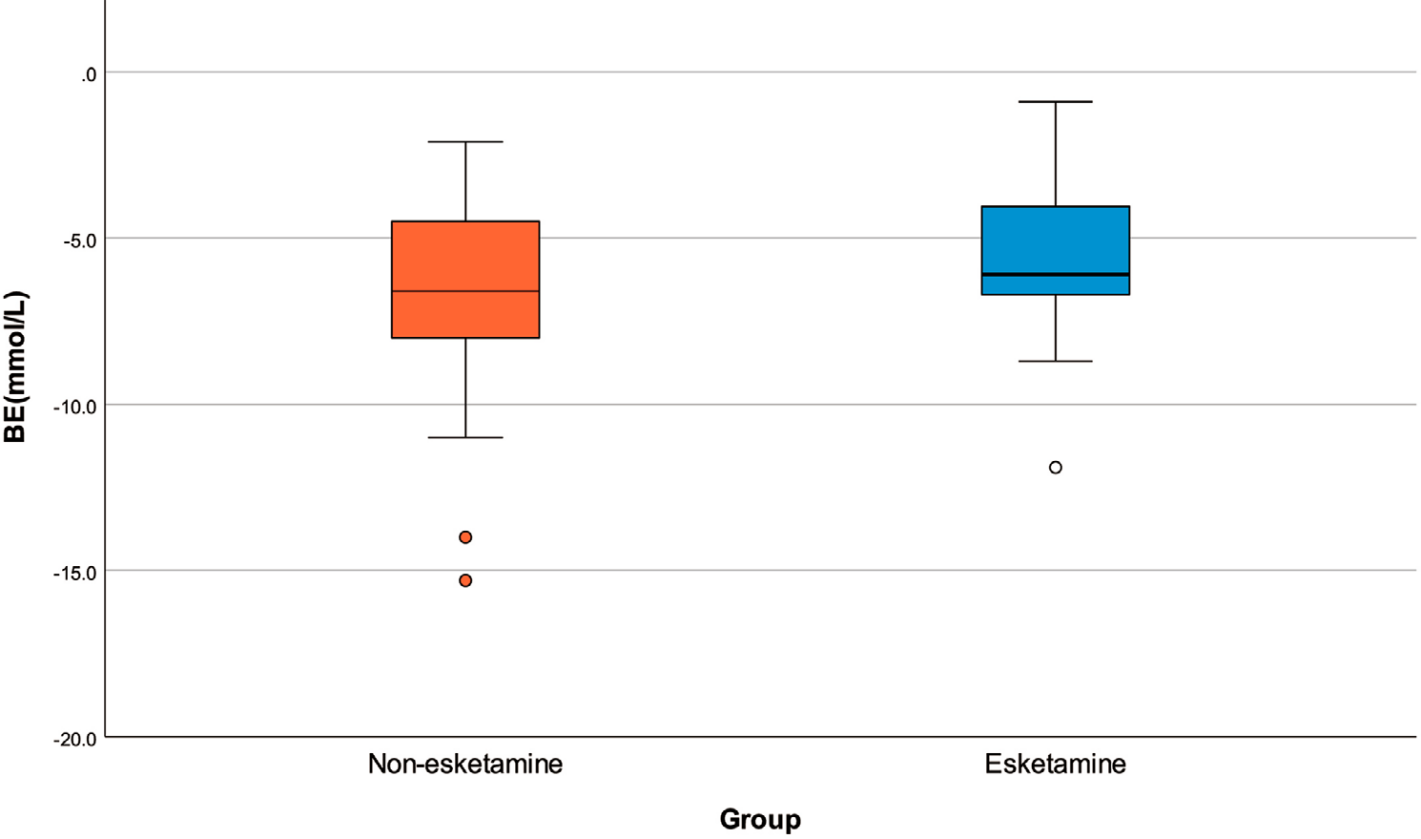
Boxplot depicting the distribution of umbilical-artery base excess in both groups

Umbilical-artery pH, BE, and lactate levels were comparable between the two groups (Table 2). BE in the esketamine group was − 5.54 ± 2.38 mM, versus − 6.63 ± 3.21 mM in the non-esketamine group; although BE was slightly lower in the esketamine group, the difference was not statistically significant (*P* = 0.186). The distribution of BE levels in each group is shown in the boxplot in Fig. 2. The incidence of fetal acidosis was 51.6% in the esketamine group and 54.8% in the non-esketamine group, which was not a statistically significant difference (*P* = 0.799).

There were no differences between the two groups in Apgar score at 1, 5, or 10 min (Table 2). Six neonates in the esketamine group but only two in the non-esketamine group had Apgar scores < 8 at 1 min (19.3% vs. 6.4%, respectively; *P* = 0.255). All neonates had Apgar scores ≥ 8 at 5 and 10 min.

**Table 2:** Comparison of neonatal outcomes

	Non-esketamine (n = 31)	Esketamine (n = 31)	*P*
UA blood gases			
pH	7.19 ± 0.08	7.23 ± 0.07	0.052
BE (mmolL^− 1^)	−6.63 ± 3.21	−5.54 ± 2.38	0.186
Lactate	4.10 ± 1.57	3.50 ± 1.19	0.130
Apgar score			
1 min	8.81 ± 0.53	8.47 ± 0.87	0.064
5 min	9.97 ± 0.17	9.91 ± 0.39	0.545
10 min	10.00 ± 0.00	9.97 ± 0.17	0.317
Total days in the hospital	4.77 ± 1.08	4.71 ± 0.90	0.869

UA = umbilical artery; BE = base excess

Two neonates in the esketamine group and one in the non-esketamine group had Apgar scores < 8 at 1 min and umbilical-artery pH < 7.20 (6.4% vs. 3.2%, respectively; *P* = 1.000).

There were no differences between the two groups in total days spent by the neonate in the hospital (Table 2); this value was 4.71 ± 0.90 in the esketamine group versus 4.77 ± 1.08 in the non-esketamine group (*P* = 0.869).

**Fig. 3:**
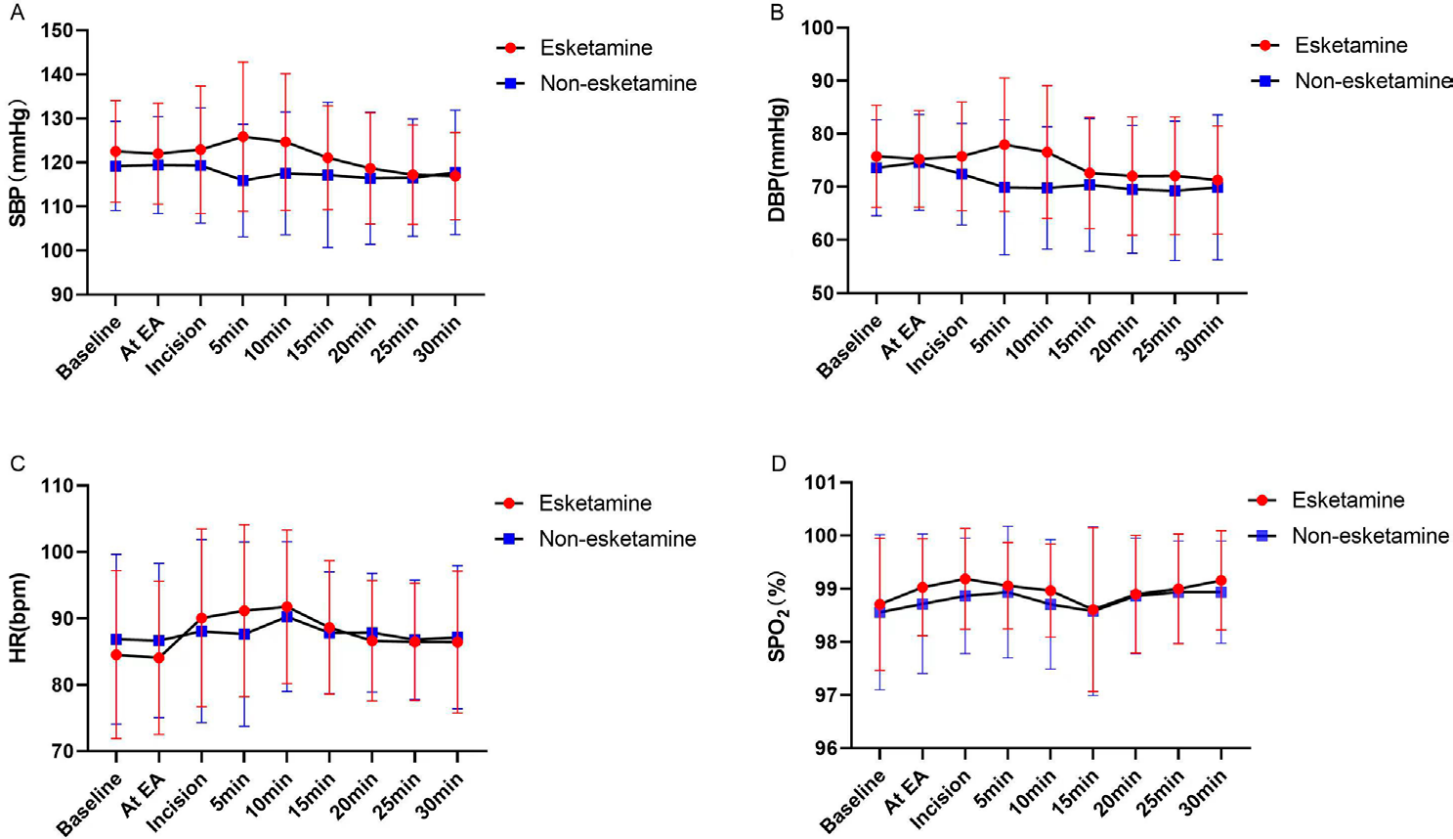
The results of repeated measurements of hemodynamic parameters. Systolic blood pressure(**A**), diastolic blood pressure(**B**), heart rate(**C**), and pulse oximetry(**D**) of parturients in two groups

All the maternal parameters were measured at baseline, EA, incision and 5 min, 10 min,15 min, 20 min, 25 min, 30 min. Compared with the non-esketamine group, SBP and DBP at 5 and 10 min were significantly higher in the esketamine group (Fig. 3). To verify whether these parameters would change over time, we tested the time*group interaction term using a repeated measures analysis of variance. Only the time*group in SBP was statistically significant (*P* = 0.035), suggesting that the trends of SBP would differ over time between the two groups. In contrast, the overall group effect revealed that there were no significant differences in maternal SBP (*P* = 0.193), DBP (*P* = 0.136), HR (*P* = 0.975) and SPO_2_ (*P* = 0.250) between the two groups.

There were no serious adverse events found in parturients. Psychic symptoms and dizziness were observed in the esketamine group and not in the non-esketamine group, but the symptoms were short-lived and not statistically significant. Other adverse events including hypotension, arrhythmia, shivering, nausea and vomiting did not differ significantly between the two groups (Table 3).

**Table 3:** Adverse events

	Non-esketamine(n = 31)	Esketamine(n = 31)	*P*
Hypotension	0	0	1.000
Arrhythmia	0	0	1.000
Nausa and voimiting	3	4	1.000
Dizziness	0	2	0.492
Shivering	3	2	1.000
Psychic symptoms	0	4	0.113

## Discussion

The use of intravenous analgesics during emergency cesarean section may lead to adverse neonatal outcomes. In our study, we observed no significant differences in neonatal outcomes between the esketamine and non-esketamine groups, including umbilical arterial-blood gas analysis, Apgar score, and total days spent by the neonate in the hospital. Additionally, our study showed a similar hemodynamic performance in parturients between the two groups during operation.

A woman who receives an emergency cesarean section may have a more negative birth experience, greater anxiety, and an increased risk of post-traumatic stress disorder (PTSD) than a woman who has an elective cesarean Sects.  [8–10] Therefore, we must pay attention to the quality of perioperative analgesia during emergency cesarean sections. At the same time, neonatal safety must be ensured before parturients receive any intervention. The analgesic of choice should have minimal transfer across the placental barrier and minimal or no side effects on the fetus.

Ketamine has been used to provide anesthesia and analgesia for decades. It can be transferred to the neonate via the placenta, [11] and its adverse effects are dose-dependent. The recommended dose of ketamine is 1–1.5 mg/kg. A subanesthetic dose of ketamine is defined as < 1 mg/kg i.v. Even a subanesthetic dose of ketamine might be able to block central sensitization [12, 13]. Because it has fewer side effects, such a dose can be administered in clinical practice. Ketamine is a racemic mixture of 50% R (−)-ketamine and 50% S (+)-ketamine. As the S + isomer of ketamine, esketamine possesses similar pharmacological characteristics but causes fewer adverse effects. Given the 2:1 potency ratio of ketamine/esketamine, the dose of esketamine should be 0.5–0.75 mg/kg. In this study, we selected a single i.v. dose of 25 mg esketamine for parturients weighing 67 kg on average, and the dose used in our study was in the safe range.

In terms of maternal hemodynamics, SBP and DBP at 5 and 10 min were significantly higher in the esketamine group in comparison with the non-esketamine group. There may be a slight increase in BP caused by esketamine. However, the overall group effect revealed that there were no significant differences in maternal SBP, DBP, HR and SPO_2_ between the two groups. To evaluate the safety of esketamine, We assessed some adverse effects including hypotension, arrhythmia, nausea and vomiting, dizziness, shivering, and psychic symptoms. Psychic symptoms and dizziness were observed in the esketamine group and not in the non-esketamine group, but the symptoms were short-lived and not statistically significant. As mentioned above, esketamine causes fewer adverse effects than ketamine and its adverse effects are dose-dependent. Accordingly, the dose of esketamine used in our study was safe for mothers.

Acute fetal acidosis is the most commom manifestation of fetal distress, and it usually occurs during delivery. Basically, it is caused by a disturbance of the maternal-fetal gas exchange. In such circumstances, there are many factors that may lead to fetal acidosis in utero. These factors include problems with the placenta and umbilical cord, excessive uterine contractions, hemodynamic instability in the mother, or anesthetic interventions. Thus, we ruled out some parturients with placental and umbilical cord problems. In the meantime, we did not observe any significant diferences in hemodynamic characteristics between the two groups during operation. As a decrease in blood pressure may compromise uterine blood flow and foetal circulation, leading to hypoxia and acidosis in the fetus [14].

Brambrink AM found that it was sensitive to the apoptotic effects of ketamine in the developing rhesus macaque brain at both fetal and neonatal periods; an exposure duration of 5 h was sufficient to cause significant neuronal apoptosis [15]. However, briefer periods of fetal exposure were not studied. In this regard, S (+)-ketamine has been shown to have neuroprotective efficacy after axonal transection and glutamate exposure in polarized rat hippocampal neurons 7 days post-insult [16]. It is not clear how to account for these contrasting results. Differences in dose, timing of administration, and the type of ketamine might contribute to the different results. Hence, more research is needed on the use of ketamine and its isomer in neonates. Based on our clinical study, we found no difference in neonatal outcomes between our two groups.

Since 1952, Apgar score has been widely used to evaluate the parturient environment of the fetus [17]. Apgar score includes heart rate, respiration, color, muscle tone, and reflexes. It can help the doctor quickly judge the neonate’s clinical status and decide on the next steps. Apgar score assessment can be biased by the evaluator’s experience and subjectivity even though there is a single standard. Furthermore, a number of factors can affect the score, including low gestational age, congenital malformations, intrauterine infection, and overuse of anesthesia. It is therefore inappropriate to diagnose asphyxia neonatorum using the Apgar score alone.

Recently, UABGA was acknowledged as the most reliable index for assessing fetal oxygenation and acid–base status. According to the American College of Obstetricians and Gynecologists (ACOG), pH < 7.00 or BE < − 12 mM in UABGA indicate significant perinatal morbidity and poor prognosis [18]. UABGA can detect even subtle effects of anesthesia on neonates, but its results can be affected by factors such as the timing of sample and test, the way of delivery. In our study, BE in the esketamine group was − 5.54 ± 2.38 mM, versus − 6.63 ± 3.21 mM in the non-esketamine group. Despite the slightly lower BE in the esketamine group, the difference was not statistically significant. BE levels among our participants were higher than those found in a previous study: −0.5 ± 1.6 mM in the supine group, and − 0.6 ± 1.5 mM in the tilt group [19]. This discrepancy might have been due to the difference in participants; that study enrolled parturients scheduled for elective cesarean section, whereas our subjects had been given labor analgesia, failed to deliver their neonates, and turned into cases of emergency cesarean section.

UABGA combined with Apgar score can reflect the metabolic state of the fetus during delivery and predict the risk of adverse events. Neonates with an Apgar score of ≤ 7 at 1 min and an umbilical-artery pH of < 7.20 can be diagnosed with mild asphyxia. In our study, two cases of mild asphyxia were recorded in the esketamine group, while one case was in the non-esketamine group. This difference between the two groups did not reach statistical significance. Furthermore, compared with the non-use of esketamine, the use of esketamine did not prolong the total days that neonates stayed in the hospital.

There were several limitations to our study. First, we did not collect concentrations of esketamine in maternal or fetal serum to provide more potent evidence. Second, we only paid attention to perioperative outcomes; the risks of long-term adverse effects, such as delayed neurological symptoms, remain unknown. Third, there was the potential to mistake umbilical venous blood for an arterial sample. The pH of umbilical arterial blood was lower than that of venous blood by at least 0.02 [20]; therefore, we might have had some errors in data analysis.

## Conclusion

In summary, intravenous esketamine (25 mg) is safe for neonates when it is given to parturients transferred from labor analgesia to emergency cesarean section. This retrospective study can provide a potential basis for drug safety in parturients during this urgent surgery.

## Data Availability

The data that support the findings of this study are available from the corresponding author upon reasonable request.
